# Inter‐annual and inter‐species tree growth explained by phenology of xylogenesis

**DOI:** 10.1111/nph.18195

**Published:** 2022-05-26

**Authors:** Yizhao Chen, Tim Rademacher, Patrick Fonti, Annemarie H. Eckes‐Shephard, James M. LeMoine, Marina V. Fonti, Andrew D. Richardson, Andrew D. Friend

**Affiliations:** ^1^ Department of Geography University of Cambridge Cambridge CB2 3EN UK; ^2^ School of Informatics, Computing, and Cyber Systems Northern Arizona University Flagstaff AZ 86011 USA; ^3^ Center for Ecosystem Science and Society Northern Arizona University Flagstaff AZ 86011 USA; ^4^ Harvard Forest Harvard University Petersham MA 01366 USA; ^5^ Institut des Sciences de la Forêt Tempérée Université du Québec en Outaouais Ripon QC JOV1V0 Canada; ^6^ Swiss Federal Research Institute for Forest, Snow and Landscape Research WSL Birmensdorf CH‐8903 Switzerland; ^7^ Department of Physical Geography and Ecosystem Science Lund University Lund S‐223 62 Sweden; ^8^ Institute of Ecology and Geography Siberian Federal University Svobodny pr 79 Krasnoyarsk 660041 Russia

**Keywords:** cell enlargement, diffuse porous, inter‐annual variability, nonstructural carbon hydrate, ring porous, temperate forest, wood phenology, xylogenesis

## Abstract

- Wood formation determines major long‐term carbon (C) accumulation in trees and therefore provides a crucial ecosystem service in mitigating climate change. Nevertheless, we lack understanding of how species with contrasting wood anatomical types differ with respect to phenology and environmental controls on wood formation.
- In this study, we investigated the seasonality and rates of radial growth and their relationships with climatic factors, and the seasonal variations of stem nonstructural carbohydrates (NSC) in three species with contrasting wood anatomical types (red oak: ring‐porous; red maple: diffuse‐porous; white pine: coniferous) in a temperate mixed forest during 2017–2019.
- We found that the high ring width variability observed in both red oak and red maple was caused more by changes in growth duration than growth rate. Seasonal radial growth patterns did not vary following transient environmental factors for all three species. Both angiosperm species showed higher concentrations and lower inter‐annual fluctuations of NSC than the coniferous species.
- Inter‐annual variability of ring width varied by species with contrasting wood anatomical types. Due to the high dependence of annual ring width on growth duration, our study highlights the critical importance of xylem formation phenology for understanding and modelling the dynamics of wood formation.

## Introduction

Wood formation determines the long‐term carbon (C) accumulation in trees and therefore provides a crucial ecosystem service in mitigating climate change. Differences in wood formation processes, for example timing, duration, and rates, and their climatic sensitivities have major influences on the assessment of global change impacts on terrestrial carbon sequestration (Rathgeber *et al*., 2016).

Current knowledge on wood formation has mainly been acquired from studies monitoring annual ring formation in conifer species (Rossi *et al*., 2006b; Cuny *et al*., 2013). The formation of wood in conifers involves multiple phases of cell development including cell division, enlargement, wall thickening and maturation (Plomion *et al*., 2001; Rathgeber *et al*., 2016), which can occur at different periods and rates depending on the climate responses of the species. The occurrences of these processes are usually confined within a growing season and constrained by the availability of resources, for example warmth and soil water, with maximum growth rate usually observed to coincide with the maximum seasonal temperature or the maximum day length (Rossi *et al*., 2006a; Cuny *et al*., 2013). The activation of cambial activity in extratropical regions is regulated by temperature and photoperiod to avoid frost damage (Rathgeber *et al*., 2016; Oribe & Funada, 2017). Recent studies also identified critical temperature and soil water potential thresholds below which radial growth does not occur (Parent *et al*., 2010; Eckes‐Shephard *et al*., 2021; Peters *et al*., 2021). Variations in a specific environmental condition above these thresholds may enhance or slow down radial growth during a growing season (Grabner *et al*., 2006; Oberhuber & Gruber, 2010; Cabon *et al*., 2020).

However, conifers are only one component of forests globally and their growth dynamic might differ from other tree types. For example, species with contrasting wood anatomical types exhibit different physiological needs, for example in the way they rely on hydraulic transportation, to adapt to the external environment. A well known example is that ring‐porous species grow faster radially in the early growing season than diffuse‐porous and coniferous species, due to their need to produce new vessel conduits each year (Garcia‐Gonzalez *et al*., 2016). These differences have also been observed to be reflected in different onset and cessation timings of xylem activities between coexisting coniferous and deciduous species (Martinez del Castillo *et al*., 2016; del Castillo *et al*., 2018). Moreover, a recent study suggested that the peak radial growth rate of ring‐diffuse species is synchronised with high soil water availability (D'Orangeville *et al*., 2022), rather than the maximum temperature or day length for coniferous species. Evidence indicates that wood formation in species with different wood anatomies is under diverse environmental, phenological and physiological controls, with impacts on intra‐ and inter‐annual ring width variations.

As the substrate and energy provider for wood formation, nonstructural carbohydrates (NSC) are expected to reflect well how tree species differently mediate the response of radial growth to environmental factors. Functionally, NSC are considered to buffer the deficits between the demands (maintenance and growth) and supply (Dietze *et al*., 2014). The dependence of wood formation on NSC should vary among species depending on how the xylem water transport is coordinated with foliage formation. At the beginning of a growing season, reinitialisation of wood formation is thought to occur earlier than bud break in ring‐porous species due to the need to rebuild earlywood vessel conduits to support water transport to the leaf buds (Wang *et al*., 1992; Takahashi *et al*., 2013; Pérez‐de‐Lis *et al*., 2018). With the absence or near absence of photosynthesis during this stage, the stored C is crucial to provide sufficient energy and material (Pérez‐de‐Lis *et al*., 2017). By contrast, the onset of wood formation of diffuse‐porous trees is less associated with C storage, as cell enlargement generally begins at or right after budbreak (Schmitt *et al*., 2000; Čufar *et al*., 2008). The onset of wood formation of coniferous species can occur either before or after bud break (Rossi *et al*., 2009). NSC concentrations of stems were observed to fluctuate during the growing season for both ring‐porous and diffuse‐porous trees under different climate and site conditions (Michelot *et al*., 2012; Richardson *et al*., 2013; Scartazza *et al*., 2013). By contrast, total NSC concentrations in stems of coniferous trees tend to accumulate first but begin to decline around the middle of the growing season (June or July), potentially caused by lower photosynthesis rate (Martínez‐Vilalta *et al*., 2016; Furze *et al*., 2019). At the end of a growing season, the NSC of both ring‐porous and diffuse‐porous trees accumulates, probably to ensure winter survival (but see Hoch *et al*. (2003) as an exception), but an opposite trend was widely observed for the coniferous trees (Martínez‐Vilalta *et al*., 2016). Such differences in the seasonal dynamics of NSC among wood types might provide key insights into different C allocation strategies with important implications for the modelling of source and sink controls over wood formation.

These diverse coordinations of phenologies and C usage among wood structures could induce differential climate sensitivities of wood formation that would significantly affect any assessment of forest C sink capacity in the context of climate change. Although wood formation within deciduous species has received some attentions in recent studies (Michelot *et al*., 2012; Kraus *et al*., 2016; Pérez‐de‐Lis *et al*., 2017; Gričar *et al*., 2022), few of them have comprehensively linked intra‐ and inter‐annual NSC variations with ring formation dynamics, and none of them has focused on differences between coexisting species with contrasting wood anatomical types.

In this study, we monitored xylogenesis, foliage phenology and NSC dynamics in *Quercus rubra* L. (northern red oak, red oak for short, a ring‐porous species), *Acer rubrum* L. (red maple, a diffuse‐porous species), and *Pinus strobus* L. (eastern white pine, a coniferous species) at a mixed temperate forest during the 2017–2019 growing seasons. We investigated the patterns and environmental drivers of annual radial growth for each species and their relationships with stem C storage using observations across a consecutive 3 yr. Our aim was to understand how intra‐annual processes, for example the timing, duration and rate of growth, generated inter‐annual variability of ring width and determine the drivers across tree species with contrasting wood anatomies. Specifically, we hypothesised that (**H_1_**) the relative importance of physiological and phenological terms to annual ring width development is wood anatomical‐type specific, and (**H_2_**) the primary environmental factors driving radial growth rate are also dependent on wood anatomies. We further investigated the variations of seasonal and inter‐annual NSC concentrations to explore their potential linkages with wood formation dynamics. we hypothesised that (**H_3_**) both seasonal and inter‐annual variations in NSC concentrations are different between species with varying wood anatomical types.

## Materials and Methods

### The study site

Harvard Forest is a mesic temperate mixed forest dominated by red oak (*Quercus rubra* L.) and red maple (*Acer rubrum* L.), with hemlock (*Tsuga canadensis*) and white pine (*Pinus strobus* L.) as the most abundant conifers. It is located in central Massachusetts, USA (42.51°N, 72.22°W). Soils at Harvard Forest are on average 1 m deep and mainly constituted of well draining, slightly acidic sandy loam. The recorded mean annual temperature and mean total annual precipitation are 7.57 ± 0.78°C (*µ ± σ*), and 1138.6 ± 227.2 mm (*µ ± σ*), respectively (Boose & Gould, 2022).

The data were collected in the Prospect Hill Tract, which has regenerated naturally since a stand‐replacing hurricane in 1938. Our study trees were located along Prospect Hill Road at an average elevation of 355 m. Initially, we selected eight codominant trees for each species: red oak, red maple and white pine. Two red maple and two white pines of the initial selection had snapped or suffered substantial damage during a storm event in 2017. We excluded data from those trees from our analysis leaving us with eight oaks, six maples and six white pines. The trees had an average age of 69 ± 6, 75 ± 15 and 81 ± 9 yr, an average height of 21.5 ± 2.1, 23.8 ± 1.9 and 25.3 ± 3.6 m, and average diameter at breast height of 25.0 ± 4.1, 47.8 ± 16.0 and 48.7 ± 15.2 cm for red maple, red oak and white pine, respectively (*µ ± σ*).

### Data collection and processing

#### Climate data

Two datasets from Harvard Forest were used to quantify the site’s long‐term climate condition. Daily temperature and precipitation during 1964–2002 and 2003–2019 were collected from Shaler Meteorological station (Boose & Gould, 2013) and the Fisher Meteorological station (Boose, 2006) at Harvard Forest, respectively. Both stations are a few hundred metres from our observational site. The potential evapotranspiration (PET) during 2001–2019 was taken from MODIS16A2 (Running *et al*., 2019). The respective spatial and temporal resolutions were 500 m and 8 d, respectively. Values of the pixel at the site and its neighbouring pixels, that is the surrounding 8 pixels, were averaged to represent the site condition. Average conditions for each month and growing season (April to September) were quantified. The ratio of precipitation to PET (P/PET) of each month and growing season was then calculated. The PET dataset was obtained and processed through Google Earth Engine (earthengine.google.com).

#### Xylogenesis

To monitor weekly growth dynamics throughout the 2017, 2018 and 2019 growing seasons, we collected microcores using a trephor tool (Rossi *et al*., 2006b). In 2017, the microcores were sampled every week from day of year (doy) 67–305. During subsequent years, the sampling period was better calibrated to only cover each species’ growing period. The sampling was then taken from doy 94–304, doy 115–283 and doy 108–304 for red oak, red maple and white pine in 2018, respectively. The corresponding periods for 2019 were from doy 128–240, doy 107–269 and doy 107–289, for red oak, red maple and white pine, respectively. Freshly sampled microcores were immediately put into Eppendorf tubes containing a 3 : 1 solution of ethanol and glacial acetic acid, which was replaced by 75% ethanol after 24 h. Using a rotary microtome (Leica RM2245; Leica Biosystem, Nußloch, Germany), we cut microsections (7 µm‐thick cross‐sectional cuts) from paraffin‐embedded samples (Tissue Processor 1020 Leica; Leica Biosystem). All samples were double stained with astra‐blue and safranin and images were produced using a digital slide scanner (Zeiss Axio Scan.Z1; Carl Zeiss AG, Jena, Germany). For each image, three radial files were chosen and the total ring width as well as zone widths for each development stage (cell division, cell enlargement, cell‐wall thickening and mature xylem cells) were measured according to Rossi *et al*. (2006a). Dates of onset and cessation of each developmental phase, that is enlargement, wall thickening and onset of maturation, were determined with the R package caviar (v.2.10‐0; Rathgeber *et al*., 2011a, 2018). These dates were defined for each species when at least 50% of the counted radial files showed the target phase. The target phase was defined according to the criteria given in Rossi *et al*. (2006a) for conifer tracheids. These criteria have been similarly applied for fibre cells in both ring‐porous and diffuse‐porous species, and using the width of the tangential band to assess its radial progress (please also refer to Prislan *et al*., 2013; Gričar *et al*., 2020). To estimate the final annual ring width, observations of xylogenesis from images after the cessation of the enlargement phase were averaged for each individual tree.

#### Foliage phenology

To identify the onset and cessation dates of foliage activity, foliage phenology of the observational trees was monitored during 2017–2019. Throughout all 3 yr we determined key phenological dates visually using the method of O'Keefe (2019). Briefly, we observed the crown of each tree with binoculars on a daily basis to determine the percentage of branches on which a phenological event (i.e. bud burst, foliage elongation, foliage coloration, and foliage fall) had occurred. We then determined a single date for each tree from when the individual process had occurred for half of the canopy of any particular tree.

#### Intra‐annual variation in NSC

To monitor intra‐annual NSC storage dynamics, stem tissues were collected in April, July and October during the 3 yr of the study, using a standard increment corer (5.15 mm diameter; Haglf Co. Group, Långsele, Sweden). The measuring dates were 5 April 2017, 5 July 2017 and 4 October 2017; 23 April 2018, 11 July 2018 and 10 October 2018; and 10 April 2019, 3 July 2019 and 2 October 2019. All samples were immediately shock frozen on dry‐ice in the field and brought to a freezer (maximum temperature of −60°C) for storage within 2 h of collection. After storage, all samples were freeze dried (FreeZone 2.5; Labconco, Kansas City, MO, USA and Hybrid Vacuum Pump, Vaccubrand, Wertheim, Germany), ground in a Wiley mill with a mesh 20 (Thomas Scientific Wiley Mill, Swedesboro, NJ, USA), and homogenised (SPEX SamplePrep 1600; MiniG, Metuchen, NJ, USA). Particularly small samples were ground with an agate pestle and mortar (JoyFay International LLC, Cleveland, OH, USA) to minimise loss of material. We homogenised the first (not including the bark and phloem) centimetre of xylem tissue. Here, *c*. 40 mg of finely ground and dried powder for each sample were analysed using a colorimetric assay with phenol–sulfuric acid following ethanol extraction, according to the protocol by Landhäusser *et al*. (2018). Absorbance values were read twice using a spectrophotometer (Genesys 10S UV‐Vis; Thermo Fisher Scientific, Waltham, MA, USA) at 490 nm for sugar and 525 nm for starch. For the quality control, we included at least eight blanks – both tube and sample blanks – and between 7 and 16 laboratory control standards (red oak stem wood, Harvard Forest, Petersham, MA, USA; potato starch, Sigma Chemicals, St Louis, MO, USA) with each batch of samples. The coefficient of variation for the laboratory control standards was 0.07 and 0.09 for sugar and starch concentrations in oak wood, respectively, and 0.13 for potato starch. To convert the sample absorbance values to concentrations in % dry weight and uncertainties we used the in‐house R package nscprocessr (https://github.com/TTRademacher/NSCprocessR) that calibrates absorbance values with a 1 : 1 : 1, glucose : fructose : galactose (Sigma Chemicals, St Louis, MO, USA) standard curve for sugar and a glucose (Sigma Chemicals) standard curve for starch. Total stem NSC concentration was then calculated as the sum of total stem soluble sugar and stem starch concentrations. These data of xylogenesis, foliage phenology, and NSC are publicly available on the Harvard Forest Data Archive (Rademacher, 2021).

#### Calculation of growth degree‐days (GDD)

To investigate cell enlargement onset in relation to temperature, we quantified GDD using the following equation:

(Eqn 1) $$\text{GDD}=\sum_{i=1}^{m}\left(T_{i}‐T_{\text{base}}\right)$$

where *T_i_* is the mean air temperature (°C) on the ith day of the year, and where m is the days with a temperature higher than the base or threshold temperature (*T*_base_, °C). We set three different *T*_base_ representing a general value range of temperate forests (2.5, 5, 7.5°C) to calculate GDD.

### Statistical analysis

General additive models were used to fit the raw xylogenesis data in different cellular development phases, that is enlargement, wall thickening and mature zones, for each individual tree following Cuny *et al*. (2013). The fitted models were then applied in the following statistical analysis.

Linear mixed effects models were used as the major statistical tool to analyse the relationships between key variables and to identify differences between years and species. To test species‐specific variations of dates of wood and foliage phenology, we set year and species as the categorical fixed effects. To test the seasonal variations of NSC terms, that is soluble sugar (SS), starch, and total NSC (SS + starch), we set observation date, year and species as the categorical fixed effects.

To test how the variations of radial growth rates responded to the transient environmental factors, we set the weekly width of enlargement zone as the response variable and tested its relationship with the weekly mean day length (DL), air temperature (*T*_a_), and precipitation (Prep), and two categorical predictors, year and species. To reduce the complexity of variable combinations, we built models using single species as an initial test to select the important environmental factors. To test the relative importance of physiological and phenological terms in determining the annual ring width, we set predictors to represent physiological and phenological conditions, that is mean and maximum weekly radial growth rates (*G*_mean_ and *G*_max_) as the physiological terms, and duration of radial growth (*G*_len_) as the phenological term, and two categorical predictors, year and species, as the fixed effects. Here, *G*_max_ and *G*_mean_ were quantified as the maximum and mean widths of the enlargement zone, respectively. *G*_len_ was calculated as the duration of cell production, that is the days between the start and end of cell enlargement. In all the above analysis based on linear mixed effect models, the tree‐specific random intercept was considered. Fixed effects were tested through setting up models with each individual component or multiple components. All models were fitted by maximum likelihood using the lmertest package in R (v.3.1‐3, Kuznetsova *et al*., 2017). Models were ranked according to their corrected Akaike’s Information Criterion scores (AIC_c_). AIC increments (∆AIC_c_) for each model were calculated with respect to that of the model with the lowest score, that is the best fitted model. ∆AIC_c_ > 2 was considered to be significant.

## Results

### Climatic conditions

**Fig. 1:**
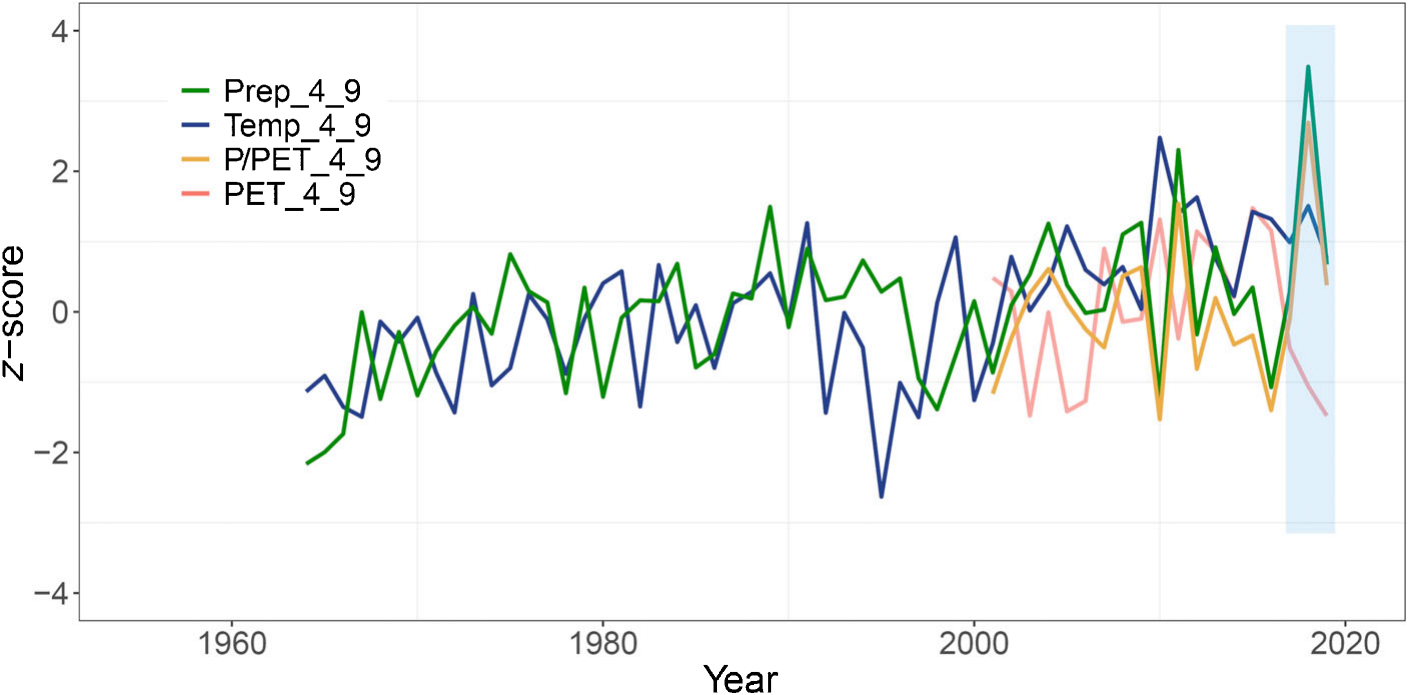
Site climate conditions during the growing season, including precipitation (Prep), temperature (Temp), potential evapotranspiration (PET), and the ratio of Prep to PET (P/PET). Results here are the average Prep, Temp, PET and P/PET for the growing season of April to September (4_9) during 1964–2019. *z*‐score is the standard score, which was calculated as the difference between the value of an index of a specific year and the multiyear mean, divided by the SD of the multiyear values. The blue shaded areas represent the study period, that is 2017–2019.

Long‐term climatic conditions of the growing season (1964–2019 for temperature and precipitation observations and 2001–2019 for PET and P/PET, respectively) at the observation site are summarised in Fig. 1. Climate indices from April to September were averaged to represent the growing season condition. The mean growing season temperatures during 2017–2019 were *c*. 1*σ* warmer than the 56‐year mean (1964–2019), respectively. The values from 2017 to 2019 were 0.98*σ*, 1.51*σ* and 0.76*σ*. The growing season precipitation of 2018 marked the highest record during the three observational years (3.48*σ* above the average), which was mainly from the high rainfall during the late growing season (July to September; Supporting Information Fig. S1). The year of 2018 also marked the rainiest year since instrumental recording. The other 2 yr were also wetter than the average conditions, with 0.28*σ* and 0.67*σ* more precipitation above the average condition. The 3 yr showed low PET with a general decreasing trend from 2017 to 2019. The lowest value was −1.48*σ* in 2019. The pattern of P/PET was very similar to that of precipitation, with significantly higher values in 2018. Additionally, it should be noticed that a relatively dry year, that is 2016, took place before the observation period. In 2016, the seasonal precipitation and P/PET of 2016 were both *c*. 1*σ* lower than the average condition.

### Inter‐annual variations of ring formation

**Fig. 2:**
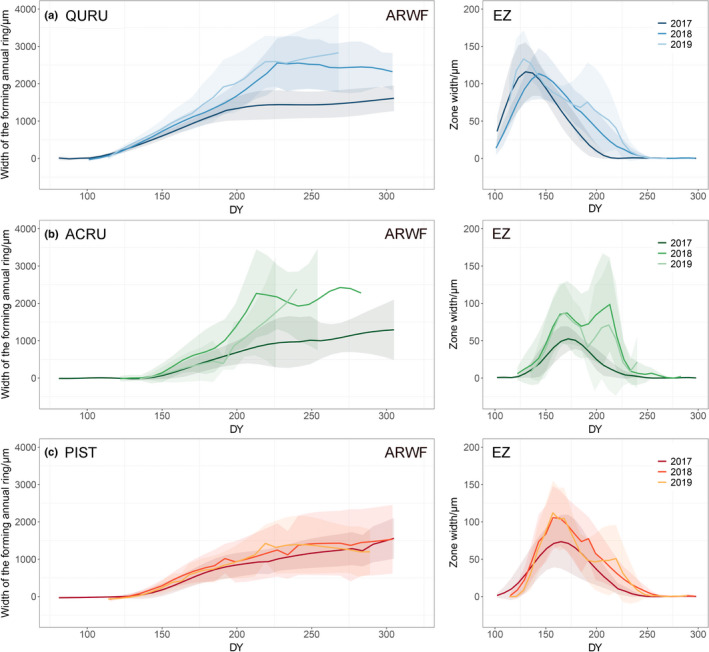
Seasonal dynamics of wood formation for three species from 2017 to 2019. The horizontal panels represent the wood formation of different species: (a) red oak (QURU), (b) red maple (ACRU), and (c) white pine (PIST). Annual ring width formation (ARWF) was calculated as the sum of the widths of cell enlargement, wall thickening and mature zones for each weekly observation. The lines represent the mean values of a specific index and the shaded areas represent ±1 SD. The sampling of red maple in 2019 was terminated early, as wood formation was observed to stop before the end date of monitoring. EZ, enlargement zone width.

Contrasting inter‐annual patterns of ring formation were found for the three species. For red oak, the mean annual ring width was significantly lower in 2017, and was 57.7% as wide as in the other 2 yr on average (59.5%, 55.9% with respect to 2018 and 2019; Fig. 2; Table S1). For red maple, higher mean ring width was also found in 2018 and 2019 but with very high variability between individuals (Table S1). Mean annual ring width of white pine barely varied during the 3 yr, with 2017 showing a slightly thinner mean ring width.

### Foliage and wood phenologies

**Fig. 3:**
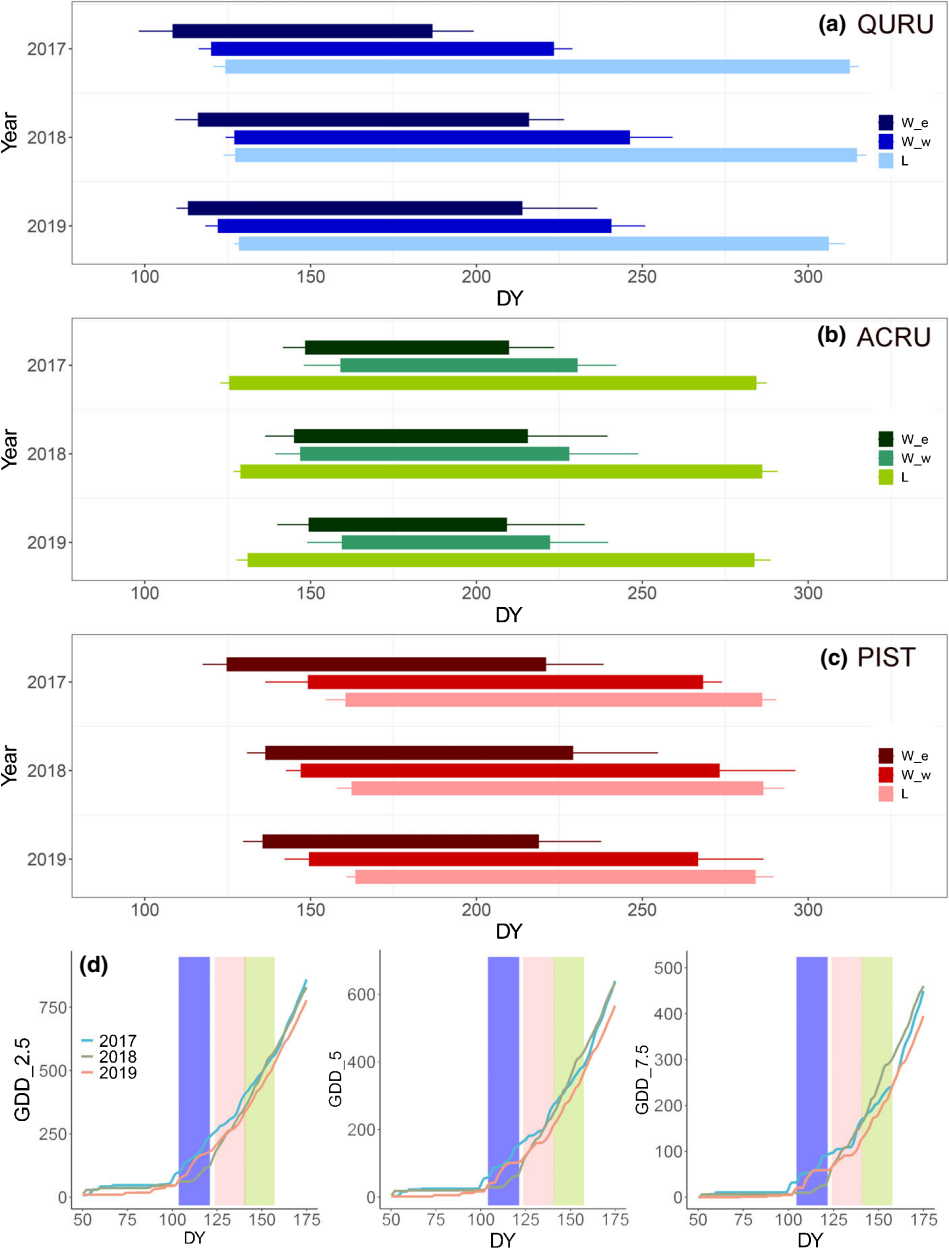
Foliage and wood growing periods of (a) red oak (QURU), (b) red maple (ACRU) and (c) white pine (PIST) during 2017–2019, defined by corresponding phenological terms. W_e, period for xylem cell enlargement; W_w, period for xylem cell‐wall thickening; L, period for foliage activity. The beginning and end of foliage activity were defined by the budburst and foliage fall, correspondingly. (d) Growth degree‐days (GDD) from 2017 to 2019 at the study site. Error bars in (a–c) represent ±1 SD. The light blue, green and red bars in (d) represent the onset of wood formation for red oak, red maple and white pine, respectively. GDD_2.5, GDD with baseline temperature of 2.5°C; GDD_5, GDD with baseline temperature of 5°C; GDD_7.5, GDD with baseline temperature of 7.5°C. DY: day of the year.

The relationship between foliage and wood phenologies for the different tree species was generally consistent across the observational 3 yr (Fig. 3). The beginning of cell enlargement was earlier than budburst for red oak and white pine, by an average of 14.7 and 29.9 d, respectively. The order was opposite for red maple, with budburst preceding the onset of cell enlargement by 18.9 d on average. Cessation dates of cell enlargement and wall thickening were much earlier than those of foliage fall for the two angiosperm species.

Using different baseline temperatures (*T*_base_), growth degree‐days (GDD) generally accumulated faster in 2017 than in the other 2 yr during the beginning period of cell enlargement for red oak (doy 112.3 ± 7.8) and white pine (doy 132.2 ± 8). After then, GDD in 2018 gradually became higher than in 2017 and 2019 during the beginning period of cell enlargement for red maple (doy 147 ± 8), especially for the calculations using 5°C and 7.5°C as *T*_base_.

Dates of budburst, foliage fall, and onset of cell enlargement showed significant differences among species and years (Table 1). With high variations between individuals, the cessation dates of enlargement and wall thickening were significantly different between years but not between species.

**Table 1:** Linear mixed models to evaluate the effect of species (red oak, red maple and white pine), and year (2017–2019) on the occurrence of principal phenological events.

Fixed effects	bE	bW	cE	cW	bb	ff	dP	dW	dF
None	41.99	34.59	3.50	20.66	95.95	59.39	4.30	21.94	93.93
Species	36.62	36.53	3.20	20.90	72.86	54.36	0.00	0.00	19.46
Year	4.02	0.00	0.12	0.00	23.27	4.92	5.73	23.93	74.20
Year + Species	0.00	1.94	0.00	0.59	0.00	0.00	1.58	1.96	0.00

The corrected Akaike’s Information Criterion increments (∆AIC_c_) for each model are shown with respect to that of the model with lowest score (the best fitted model). ∆AIC_c_ > 2 are considered to be significant. bb, budburst; bE, beginning of cell enlargement; bW, beginning of wall thickening; cE, cessation of enlargement; cW, cessation of wall thickening; dF, duration of foliage activity; dP, duration of cell production; dW, duration of wood formation; ff, foliage fall.

The mean annual duration of cell production and wood formation (cell enlargement and wall thickening) was longest for white pine and then red oak and red maple (Fig. 3; Table S1). We observed shorter durations of enlargement and wood formation in 2017 than the other 2 yr for red oak, but similar durations for red maple and white pine over the 3 yr. Through the mixed effects model, significant differences of durations were found between species but not between years.

Regarding foliage phenology, the duration of foliage activity, that is days between budburst and foliage fall, showed significant differences between both years and species (Table 1).

### Intra‐ and inter‐annual xylogenesis dynamics

As the critical phase for radial growth, cell enlargement started and peaked earlier for red oak than the other two species in all 3 yr (Figs 2, 3). The timings of peak zone width (first peak in case of red maple) were at doy 137.8 (±9.1), 172.9 (±7.0), and 163.2 (±8.4) for red oak, red maple and white pine, respectively. Individual trees from all three species exhibited a single peak pattern in 2017, with more similar timing of the peak than in the other 2 yr (Figs S2–S4). On average red maple showed a bi‐model growth pattern in 2018 and 2019, but individual trees exhibited uni‐ and bi‐modal growth dynamics (Fig. S3). The end of cell enlargement from all species showed high variabilities between individuals, ranging from doy 175 to 250.

We first tested the importance of different environmental factors, that is weekly mean daylength (DL), air temperature (*T*_a_), and precipitation (Prep) on variations in intra‐annual radial growth rate, that is weekly mean width of the enlargement zone. Based on an initial analysis within each species, we identified DL and *T*_a_ as the most significant environmental factors, and so were tested further using the two factors (Tables S2–S4). DL played a dominant role in affecting the variations of weekly radial growth rate during the 3 yr. Models involving DL showed substantial better performance than those only involving *T*_a_ (Table 2). Among the species, the weekly radial growth rate of white pine showed the closest relationship against DL, with marginal *R* ^2^ reaching 0.44 using DL as the single fixed effect (Table S4). *T*_a_ can explain a relatively smaller proportion of the variations of cell enlargement, and mainly for red oak and white pine (Tables S2–S4). Incorporating the interactions between *T*_a_ and DL could further enhance the model performance, but could only marginally increase the explanatory power for the variations of radial growth rate. No general effect of Prep was found. Its effect was more transient. One example is a synchronisation of high rainfall and growth stimulation in 2018 for red maple (Figs 2, S5).

**Table 2:** Linear mixed models to evaluate the responses of the weekly variations of enlargement zone, against day length (DL) and air temperature (*T*_a_) for the three species (red oak, red maple and white pine) during 2017–2019.

Models	Fixed effects	Variations of enlargement zone		
		ΔAIC_c_	Marginal *R^2^*	Conditional *R* ^2^
M1	None	1219.00	0.00	0.00
M2	Species	1216.00	0.018	0.049
M3	Year	1105.00	0.080	0.11
M4	DL	433.00	0.35	0.41
M5	*T*_a_	1122.00	0.053	0.11
M6	DL × Year	333.00	0.39	0.45
M7	*T*_a_ × Year	1047.00	0.94	0.15
M8	DL × Species	424.00	0.37	0.41
M9	*T*_a_ × Species	1079.00	0.094	0.13
M10	DL × Year × Species	326.00	0.41	0.45
M11	*T*_a_ × Year × Species	980.00	0.15	0.18
M12	DL × *T*_a_	408.00	0.36	0.42
M13	DL × *T*_a_ × Species	270.00	0.41	0.47
M14	DL × *T*_a_ × Year	149.00	0.46	0.50
M15	DL × *T*_a_ × Year × Species	0.00	0.51	0.55

The corrected Akaike’s Information Criterion increments (∆AIC_c_) for each model are shown with respect to that of the model with lowest score (the best fitted model). ∆AIC_c_ > 2 are considered to be significant.

Overall, the annual ring width can be better explained by the duration of radial growth (*G*_len_) than mean (*G*_mean_) or maximum (*G*_max_) weekly radial growth rates (Table 3). Models with consideration of *G*_len_ showed substantial lower AIC and higher explanatory power for annual ring width than the models considering only *G*_mean_ or *G*_max_. The varied role of *G*_mean_ and *G*_max_ was probably a consequence of the high intra‐annual variability of cell enlargement between years and individuals (Figs S2–S4). Models considering interactions between *G*_len_ and species compared favourably against models not including these interactions, indicating different responses existed between species (Tables 3, S5). The year factor showed a minor effect on the annual ring width prediction compared with that among species. For individual species, *G*_len_ played a major role in controlling the annual ring width for red oak and red maple (Tables S6–S8). For white pine, there was a strong effect on annual ring width from the individual trees (*R* ^2^ = 0.68). We also found an effect of *G*_mean_ for white pine, but it was significantly less important than the tree factor (Table S9). By contrast, *G*_max_ and *G*_len_ showed comparably minor effects on ring width growth for this species (Table S7). To further explain this strong effect from the individuals, we further tested the relationship between diameter at breast height (DBH) and mean annual ring width of individual trees, and found a significant relationship for white pine (*R* ^2^ = 0.73, *P* < 0.01), but not for the other species (Fig. S6).

**Table 3:** Linear mixed models to evaluate the effect of the maximum enlargement zone width (*G*_max_) or mean enlargement zone width (*G*_mean_), and the duration of enlargement phase (*G*_len_) on the annual ring width for the three species (red oak, red maple and white pine) during 2017–2019.

Models	Fixed effects	Variations of enlargement zone		
		ΔAIC_c_	Marginal *R* ^2^	Conditional *R* ^2^
M1	None	31.72	0.00	0.097
M2	Year	28.97	0.072	0.024
M3	Species	28.95	0.077	0.090
M4	*G*_max_	28.95	0.083	0.21
M5	*G*_mean_	21.27	0.20	0.31
M6	*G*_len_	14.95	0.31	0.54
M7	*G*_len_ × Species	1.65	0.50	0.62
M8	*G*_len_ × Year	12.43	0.36	0.59
M9	*G*_len_ × Species × Year	0.00	0.60	0.69
M10	*G*_max_ × *G*_len_	18.00	0.32	0.53
M11	*G*_mean_ × *G*_len_	14.43	0.36	0.54

The corrected Akaike’s Information Criterion increments (∆AIC_c_) for each model are shown with respect to that of the model with lowest score (the best fitted model). ∆AIC_c_ > 2 are considered to be significant. A complete output with all models we tested is listed in Supporting Information Table S5.

### Seasonal dynamics of NSC

**Fig. 4:**
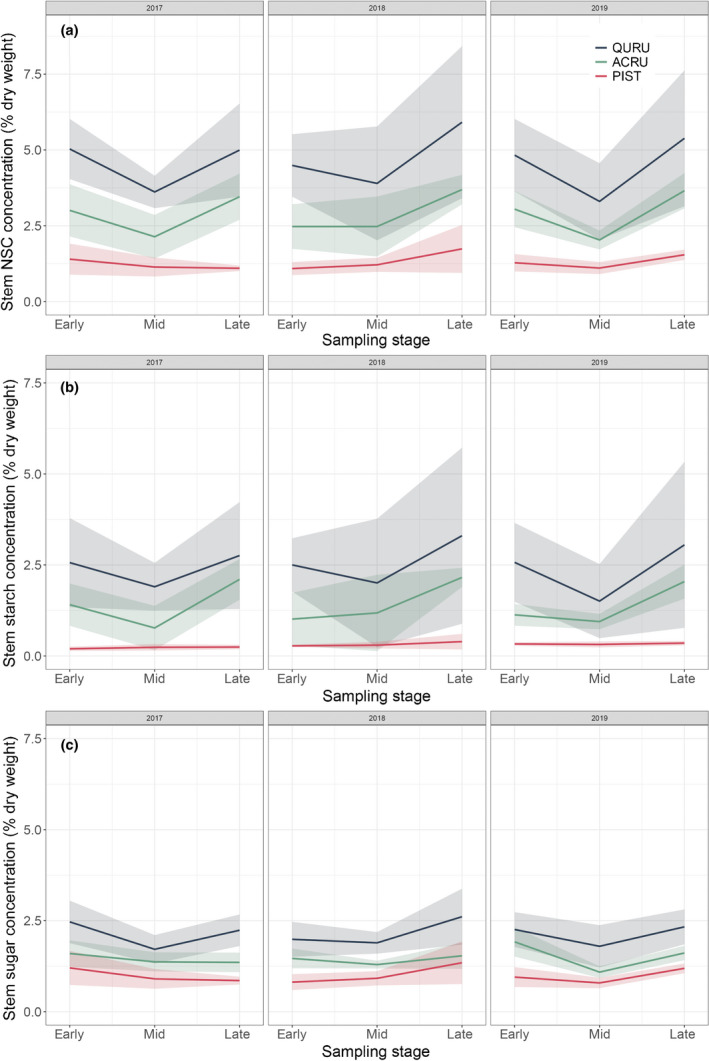
Seasonal dynamics of stem (a) total nonstructural carbohydrates (NSC), (b) starch and (c) soluble sugar for red oak (QURU), red maple (ACRU) and white pine (PIST) from 2017 to 2019. Early, mid and late denote the sampling stages in April, July and September, respectively. The shaded areas represent ±1 SD.

Concentrations were significantly different among the three species for total stem NSC, soluble sugar (SS), and starch, with all the well fitted models containing the species effect (Fig. 4; Table 4). In contrast with the obvious difference in annual ring width for red oak and red maple, both the year and observational date showed a minor effect on the stem NSC concentration terms. Seasonal variations of mean stem NSC were consistent over years for both red oak and red maple. The relatively low NSC concentrations appeared in the middle of the growing season (early July), corresponding to the middle timing of wall thickening, that is the major stage for trees to deposit C into wood. While the high NSC concentrations appeared at the early and late growing season, corresponding to the timing before and after wall thickening. For white pine, stem NSC concentrations decreased continuously from early to late growing season in 2017. The pattern was reversed in 2018, with an increasing trend throughout the growing season. In 2019, stem NSC concentration first decreased and then increased. However, due to the variations between individuals, no significant difference between years was found (Fig. S7).

**Table 4:** Linear mixed models to evaluate the effect of date, species (red oak, red maple and white pine) and year (2017–2019) on the seasonal dynamics of stem nonstructural carbohydrates (NSC).

Models	Fixed effects	Soluble sugar	Starch	NSC
		ΔAIC_c_	ΔAIC_c_	ΔAIC_c_
M1	None	27.25	22.25	30.51
M2	Date	29.09	24.12	32.30
M3	Year	29.14	24.25	32.49
M4	Species	0.00	8.01	6.36
M5	Date + Year	30.51	14.24	24.15
M6	Date + Species	1.84	9.88	8.15
M7	Year + Species	1.89	10.01	8.34
M8	Date + Year + Species	3.26	0.00	0.00
M9	Date + Year × Species	6.75	1.27	1.57
M10	Date × Year + Species	5.24	3.76	3.88
M11	Date × Year × Species	12.41	6.81	11.09

The corrected Akaike’s Information Criterion increments (∆AIC_c_) for each model are shown with respect to that of the model with lowest score (the best fitted model). ∆AIC_c_ > 2 are considered to be significant.

## Discussion

### Duration vs rate to determine annual ring width

The duration of radial growth (*G*_len_) is determined by xylem formation phenology, that is the timing of onset and cessation of cell enlargement, while the radial growth rates (*G*_max_ or *G*_mean_) reflect more the intrinsic growth potential under a specific environment. Our results confirmed **H_1_**, that is the relative importance of physiological and phenological terms to annual ring width development is wood anatomy‐type specific, by revealing the dominant role of *G*_len_ in determining annual ring width variation between years for both angiosperm species, in contrast with within white pine. Previous studies have found that variations of ring width in conifers (Vaganov *et al*., 2006; Rathgeber *et al*., 2011b; Cuny *et al*., 2012; Ren *et al*., 2019) and ring‐porous species (Delpierre *et al*., 2016; Pérez‐de‐Lis *et al*., 2017), were mainly attributed to *G*_max_ rather than *G*_len_. One exception to such a *G*_max_ dominant view is from a comparison using trees with three different wood anatomical types, in which annual ring width was significantly correlated with the end date of the growing season, but not with *G*_max_, implying a phenological control over radial growth on an annual basis over different tree types (Michelot *et al*., 2012). Through multiyear observations, this study is the first direct evidence to indicate a strong dependence of annual ring width on *G*_len_ for the typical angiosperm species under the same environmental conditions.

Regarding white pine, we observed relatively minor effects from both duration and rate terms to explain annual ring width. Instead, a major effect was generated from the tree factor. This suggests that radial growth in this species is controlled by the intrinsic biological condition, for example vitality, as a high correlation between DBH and mean annual ring width was found (Fig. S6). In addition, the observed growth patterns in pine seemed to be not simply determined by a single species‐specific strategy, as we observed individual trees of white pine with different growth patterns, for example short *G*_len_ and low *G*_max_, short *G*_len_ and medium *G*_max_, long *G*_len_ and medium *G*_max_, and so on (Fig. S4). Therefore, the outcome also reflected each individual’s fit to its corresponding micro‐physical and biological environment in an ecosystem (Rathgeber *et al*., 2011b).

### Inter‐annual variability of phenologies

The general features of foliage and wood phenologies from the different species in this study were consistent with previous observations. For all 3 yr, the onsets of wood development preceded or followed the budburst for red oak and red maple, respectively. This corroborated previous conclusions for ring‐porous and diffuse‐porous trees (Takahashi *et al*., 2013; Guada *et al*., 2020). White pine showed an earlier onset of growth than bud burst, which was similar to findings from other coniferous species (e.g. Rossi *et al*., 2009; Moser *et al*., 2010; Takahashi & Koike, 2014).

Environmental drivers of the beginning of cell enlargement are relatively well understood. Recent studies have suggested that a comprehensive consideration of photoperiod and temperature can accurately estimate the onset of radial growth of various species (Delpierre *et al*., 2019; Huang *et al*., 2020). Based on the GDD calculation, the inter‐annual variability of spring growth onset of different species from our study can be interpreted. The early spring of 2017 was warmer than the other 2 yr, leading to a corresponding faster accumulation of GDD during the onset period for red oak and white pine in 2017. By contrast, the GDD of 2018 accumulated faster than in the other 2 yr for red maple, possibly leading to a slightly earlier growth onset for that year (Fig. 3d). The relative low variabilities of spring onset between individuals further confirmed the robustness of the results of the inter‐annual variations of growth onset from the three species.

Factors determining the timing of cessation of cell enlargement, however, are not well understood in either conifers or angiosperms. Current knowledge on this phenological transition in wood formation is mainly from conifers and at the cellular or individual level, and suggests a potential combined effect from hormonal signals, resource availability, and direct environmental factors (Uggla *et al*., 2001; Sorce *et al*., 2013; Cartenì *et al*., 2018). At the individual level, inconsistent patterns of enlargement cessation were found between years, which seemed to be not controlled by any single factor (Fig. S8). Once averaged to the stand level, we identified the high variability between individuals for each species. Interestingly, we observed a potential linkage between the start and end of enlargement for red oak at the stand level, which was similar to the findings from foliage phenology (Fu *et al*., 2014; Keenan & Richardson, 2015; Zani *et al*., 2020). The early cessation of cell enlargement for red oak in 2017 corresponded to the earlier beginning of cambial activity and later cessation seems linked to the delay of onset for the other 2 yr (Fig. 3). One possible explanation for the covarying pattern is the sink limitations from nutrient supply or phloem loading (Paul & Foyer, 2001; Ryan & Asao, 2014). However, the phenomenon of earlier growth cessation has not been widely observed in the free‐air CO_2_ enrichment (FACE) experiments (Norby, 2021) and the other two species involved in this study. In addition, the preceding autumn phenology may also affect the phenology of current year by modifying the timing and duration of dormancy period (Marchand *et al*., 2021). Our results, therefore, call for future tests to better understand the underlying mechanisms driving the variations of autumn phenology, especially for species other than conifers.

### Endogenous properties, rather than transient environmental factors, control radial growth dynamics

The variations of weekly width of enlargement zone tracked photoperiod more than the other transient environmental factors at the site. Therefore, we reject **H_2_** by identifying a common primary environmental factor for all three species independent of wood anatomy. Among the three species, white pine exhibited the highest correlation with photoperiod. On the one hand, the close relationship between the variability of weekly width of enlargement zone and photoperiod in white pine suggests a relatively consistent bell‐shaped pattern of radial growth for white pine. *G*_max_ of white pine was generally reached around the summer solstice day (doy 173), confirming the conclusions of previous studies (Rossi *et al*., 2006b; Cuny *et al*., 2015). On the other hand, this relatively constant pattern over years also indicates a lower plastic response to environmental factors compared with the two angiosperm species. Regarding red oak, cell enlargement peaked earlier than for the other two species due to the requirement to form vessels at the beginning of the growing season, and therefore presents an asymmetric growth pattern with rapid width increment during the early growing season. Red maple exhibited the highest plasticity of cell enlargement among the three species. All individuals reached the first growing peak around the summer solstice (Figs 2, S3), but individuals with longer *G*_len_ exhibited a second surge of growth after the first peak in 2018 (several trees in 2019 as well). The second peak was synchronised with the high water input after July (Fig. S5). This species‐specific response is potentially related to the high sensitivity of red maple wood formation to water supply. Due to its shallow root system, water demand of red maple is strongly dependent on surface soil moisture, potentially causing variation in both foliage and wood activities (Gilman, 1990; Tschaplinski *et al*., 1998). The substantially lower ring width only for red maple in 2016, that is the year with a dry growing season, seems to corroborate this explanation (Fig. S9; Notes S1).

### Seasonal and inter‐annual variability of NSC

Species‐specific NSC concentrations clearly reflected the differential dependence on C storage by each species through the growing season. Therefore, we accept **H_3_**. The substantially higher concentration of NSC and larger magnitude of changes for red oak and red maple indicated the greater demand of C storage during different periods, for example onset of growth and dormancy period, than for the coniferous species. Our study therefore provides evidence and explanation for the substantial differences in C storage levels by linking them to the species‐specific growth strategies.

In contrast with that found for annual ring widths, no significant changes in red oak and maple NSC were observed across years, suggesting that radial growth was not always limited by C supply. In red oak, for example, the earlier onset of wood formation in 2017 did not result in lower stem NSC concentrations than in other years. This result suggested a sink limitation to wood formation, possibly induced by temperature limitation. However, we could not exclude that the seasonally constant NSC concentration was maintained by NSC remobilisation from other organs as suggested by Barbaroux & Breda (2002). As shown by Furze *et al*. (2019), there was a significant reduction in branch NSC and a corresponding increment of stem NSC during April in both red oak and maple at the same site. A recent study has also suggested that storage in living bark tissue can be a source of NSC remobilisation for xylem formation (Schoonmaker *et al*., 2021). This xylem NSC maintenance may cause C depletion in other organs and induce further feedbacks between source and sink activities (Hartmann & Trumbore, 2016). In addition, low stem NSC concentration was observed during the middle of the wall‐thickening stage for red oak and red maple. The result is different from the existing evidence for coniferous species, for which stem NSC concentration generally remained high during the similar timing of a year (Oberhuber *et al*., 2011; Simard *et al*., 2013). Our observations, therefore, implied a more significant investment of C storage in support of C deposition into wood for both ring‐porous and diffuse‐porous species than the coniferous ones. Nevertheless, due to the high variability of NSC observed in the above‐ground organs (Tixier *et al*., 2018), the frequency and timing of the NSC observation was also critical to illustrate the seasonal dynamics of C supply condition. Implementing comprehensive measurements of the whole tree NSC pattern with fine temporal resolutions will be helpful to better understand the role of C storage and the effects on wood formation.

### Conclusion

We have identified species‐specific inter‐annual variability of ring width linked to wood anatomical types and drivers. Both red oak and red maple showed a high dependence of annual ring width on the duration of radial growth (*G*_len_). For red oak, with shorter *G*_len_ in 2017, a thinner ring width of that year was identified than for the other 2 yr. For red maple, similar *G*_len_ were found for all 3 yr. We observed a larger mean ring width in 2018, potentially due to the high water supply during the late growing season. Whereas with high plasticity of cell enlargement, large differences of annual ring width between individuals were found. For white pine, ring width mainly varied between individuals rather than years, with only minor inter‐annual variability. Such diversity of wood formation activity between species at a single site suggested that an aggregated inter‐annual variability would be different from any of the single species once aggregated to the ecosystem level. This should be responsible for the lack of a clear role of transient environmental factors for intra‐annual variations of radial growth from our study.

It is important to emphasise that our current understanding of wood formation was largely derived from conifers, and model development is inevitably based on existing knowledge (Vaganov *et al*., 2006; Fatichi *et al*., 2019). With increasing recognition of the importance of sink activities, it is necessary to know how to adequately represent xylogenesis in global models across all tree anatomies (Friend *et al*., 2019). We have identified contrasting patterns of xylogenesis from trees with different wood anatomies at the same site. Our results highlighted the fact that species‐specific wood anatomical types and differences in their sensitivities to environmental pressures largely determined the annual developmental dynamics of ring width increment, with differences in timing, duration, and rate (D'Orangeville *et al*., 2022). It is therefore essential to study the drivers of the dynamics of wood formation in species other than conifers to meet future modelling requirements. Our results are a step in this direction by providing new insights into the importance of the duration of radial growth for wood formation of angiosperms, and therefore increases the understanding of the drivers of inter‐annual and inter‐species variations in wood formation.

## Author contributions

All authors contributed to the design of the study. TR, PF, JML and MVF realised the sample collection and processing. YC, TR and AHES interpreted and analysed data under guidance of ADR and ADF. YC drafted the manuscript, with inputs from all authors.

## Supporting information


**Fig. S1** The monthly climatic conditions of growing season for the study site from 1964 to 2019.
**Fig. S2** Individual level intra‐annual pattern of xylogenesis for red oak during 2017–2019.
**Fig. S3** Individual level intra‐annual pattern of xylogenesis for red maple during 2017–2019.
**Fig. S4** Individual level intra‐annual pattern of xylogenesis for white pine during 2017–2019.
**Fig. S5** Comparison of weekly precipitation of the study years against multiple year mean.
**Fig. S6** Correlations between mean annual diameter at breast height (DBH) and mean annual ring width of individual trees for red oak, red maple and white pine.
**Fig. S7** Seasonal dynamics of stem NSC for red oak, red maple and white pine of individuals from 2017 to 2019.
**Fig. S8** Dates of enlargement cessation of individuals (cE_date) for the three species.
**Fig. S9** The inter‐annual patterns of mean ring width of red oak, maple and white pine from 2016 to 2019.
**Notes S1** Quantification of annual ring width based on the standardisation data.
**Table S1** Summary of important growth indexes.
**Table S2** Linear mixed models to evaluate the responses of the weekly variations of enlargement zone, against day length (DL), air temperature (*T*_a_) and precipitation (Prep) for red oak during 2017–2019.
**Table S3** Linear mixed models to evaluate the responses of the weekly variations of enlargement zone, against day length (DL), air temperature (*T*_a_) and precipitation (Prep) for red maple during 2017–2019.
**Table S4** Linear mixed models to evaluate the responses of the weekly variations of enlargement zone, against day length (DL), air temperature (*T*_a_) and precipitation (Prep) for white pine during 2017–2019.
**Table S5** All linear mixed models were tested to evaluate the effect of the maximum enlargement zone width (*G*_max_) or mean enlargement zone width (*G*_mean_), and the duration of enlargement phase (*G*_len_) on the annual ring width for the three species during 2017–2019.
**Table S6** Linear mixed models to evaluate the effect of the maximum enlargement zone width (*G*_max_), the mean enlargement zone width (*G*_mean_) and the duration of enlargement phase (*G*_len_) on the annual ring width for red oak during 2017–2019.
**Table S7** Linear mixed models to evaluate the effect of the maximum enlargement zone width (*G*_max_), the mean enlargement zone width (*G*_mean_) and the duration of enlargement phase (*G*_len_) on the annual ring width for red maple during 2017–2019.
**Table S8** Linear mixed models to evaluate the effect of the maximum enlargement zone width (*G*_max_), the mean enlargement zone width (*G*_mean_) and the duration of enlargement phase (*G*_len_) on the annual ring width for white pine during 2017–2019.
**Table S9** Linear mixed models to evaluate the effect of the mean enlargement zone width (*G*_mean_) and the individual tree (tree) on the annual ring width for white pine during 2017–2019.
Please note: Wiley Blackwell are not responsible for the content or functionality of any Supporting Information supplied by the authors. Any queries (other than missing material) should be directed to the *New Phytologist* Central Office.

## Data Availability

The data that support the findings of this study are openly available in Harvard Forest Data Archive at https://harvardforest1.fas.harvard.edu/exist/apps/datasets/showData.html?id=HF361.
