# Supplementary material for: Inter‐annual and inter‐species tree growth explained by phenology of xylogenesis
Source: New Phytol. 2022 May 26;235(3):939–52. doi: 10.1111/nph.18195 (PMC9325364; doi:10.1111/nph.18195)
Supplement: Supplementary file 1 — Fig. S1 The monthly climatic conditions of growing season for the study site from 1964 to 2019. Fig. S2 Individual level intra‐annual pattern of xylogenesis for red oak during 2017–2019. Fig. S3 Individual level intra‐annual pattern of xylogenesis for red maple during 2017–2019. Fig. S4 Individual level intra‐annual pattern of xylogenesis for white pine during 2017–2019. Fig. S5 Comparison of weekly precipitation of the study years against multiple year mean. Fig. S6 Correlations between mean annual diameter at breast height (DBH) and mean annual ring width of individual trees for red oak, red maple and white pine. Fig. S7 Seasonal dynamics of stem NSC for red oak, red maple and white pine of individuals from 2017 to 2019. Fig. S8 Dates of enlargement cessation of individuals (cE_date) for the three species. Fig. S9 The inter‐annual patterns of mean ring width of red oak, maple and white pine from 2016 to 2019. Notes S1 Quantification of annual ring width based on the standardisation data. Table S1 Summary of important growth indexes. Table S2 Linear mixed models to evaluate the responses of the weekly variations of enlargement zone, against day length (DL), air temperature (T a) and precipitation (Prep) for red oak during 2017–2019. Table S3 Linear mixed models to evaluate the responses of the weekly variations of enlargement zone, against day length (DL), air temperature (T a) and precipitation (Prep) for red maple during 2017–2019. Table S4 Linear mixed models to evaluate the responses of the weekly variations of enlargement zone, against day length (DL), air temperature (T a) and precipitation (Prep) for white pine during 2017–2019. Table S5 All linear mixed models were tested to evaluate the effect of the maximum enlargement zone width (G max) or mean enlargement zone width (G mean), and the duration of enlargement phase (G len) on the annual ring width for the three species during 2017–2019. Table S6 Linear mixed models to evaluate the effect of the maximum en [file NPH-235-939-s001.pdf]

New Phytologist

New Phytologist Supporting Information

Article title: Inter-annual and inter-species tree growth explained by phenology of xylogenesis
Authors: Yizhao Chen, Tim Rademacher, Patrick Fonti, Annemarie H. Eckes-Shephard, James M. LeMoine, Marina V. Fonti, Andrew D. Richardson, Andrew D. Friend
Article acceptance date: 15 April 2022

The following Supporting Information is available for this article:

Fig. S1 The monthly climatic conditions of growing season for the study site from 1964 to 2019.

Fig. S2 Individual level intra-annual pattern of xylogenesis for red oak during 2017-2019.

Fig. S3 Individual level intra-annual pattern of xylogenesis for red maple during 2017-2019.

Fig. S4 Individual level intra-annual pattern of xylogenesis for white pine during 2017-2019.

Fig. S5 Comparison of weekly precipitation of the study years against multiple year mean. DY: day of year.

Fig. S6 Correlations between mean annual diameter at breast height (DBH) and mean annual ring width of individual trees for (a) red oak, (b) red maple and (c) white pine.

Fig. S7 Seasonal dynamics of stem NSC for red oak, red maple and white pine of individuals from 2017 to 2019.

Fig. S8 Dates of enlargement cessation of individuals (cE_date) for the three species.

Fig. S9 The interannual patterns of mean ring width of red oak, maple and white pine from 2016 to 2019.

Table S1 Summary of important growth indexes.

Table S2 Linear mixed models to evaluate the responses of the weekly variations of enlargement zone, against day length (DL), air temperature (Ta) and precipitation (Prep) for red oak during 2017-2019.

New Phytologist

Table S3 Linear mixed models to evaluate the responses of the weekly variations of enlargement zone, against day length (DL), air temperature (Ta) and precipitation (Prep) for red maple during 2017-2019.

Table S4 Linear mixed models to evaluate the responses of the weekly variations of enlargement zone, against day length (DL), air temperature (Ta) and precipitation (Prep) for white pine during 2017-2019.

Table S5 All linear mixed models tested to evaluate the effect of the maximum enlargement zone width (G_{max}) or mean enlargement zone width (G_{mean}), and the duration of enlargement phase (G_{len}) on the annual ring width for the three species during 2017-2019.

Table S6 Linear mixed models to evaluate the effect of the maximum enlargement zone width (G_{max}), the mean enlargement zone width (G_{mean}) and the duration of enlargement phase (G_{len}) on the annual ring width for red oak during 2017-2019.

Table S7 Linear mixed models to evaluate the effect of the maximum enlargement zone width (G_{max}), the mean enlargement zone width (G_{mean}) and the duration of enlargement phase (G_{len}) on the annual ring width for red maple during 2017-2019.

Table S8 Linear mixed models to evaluate the effect of the maximum enlargement zone width (G_{max}), the mean enlargement zone width (G_{mean}) and the duration of enlargement phase (G_{len}) on the annual ring width for white pine during 2017-2019.

Table S9 Linear mixed models to evaluate the effect of the mean enlargement zone width (G_{mean}) and the individual tree (tree) on the annual ring width for white pine during 2017-2019.

Notes S1 Quantification of annual ring width based on the standardization data

Fig. S1 The monthly climatic conditions of (a) Apr., (b) May, (c) Jun., (d) Jul., (e) Aug., (f) Sept. for the study site from 1964 to 2019, including precipitation (Prep), temperature (Temp), potential evapotranspiration (PET) and the ratio of Prep to PET (P/PET). The blue shaded area is the study period, i.e., 2017-2019.

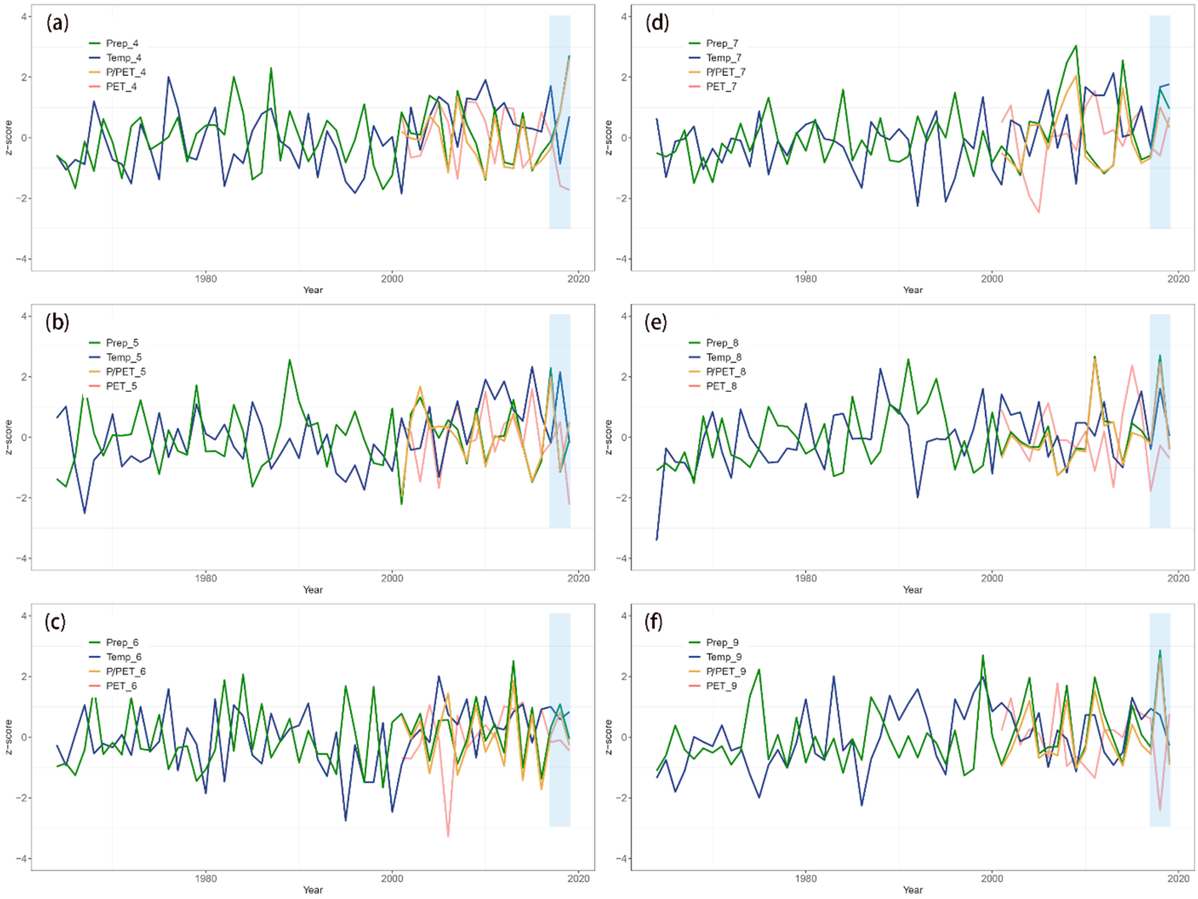

Fig. S2 Individual level intra-annual pattern of xylogenesis for red oak during 2017-2019. The points and lines correspond to the raw and fitted data using a generalized additive model, respectively. Individual trees are colour-coded (legend on the right). Zone widths for cell elongation (EZ), cell-wall thickening (WZ), and mature xylem cells (MZ) are displayed in the left, middle, and right panels, respectively.

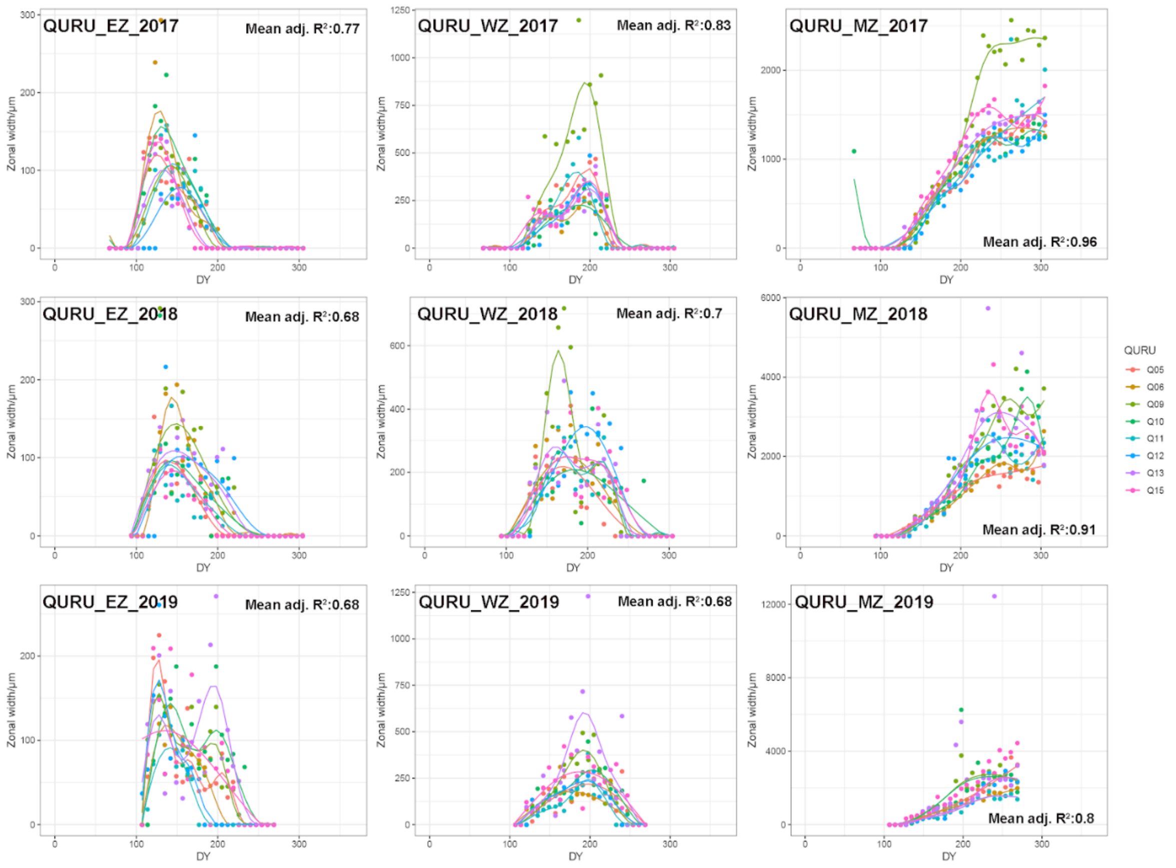

New Phytologist

Fig. S3 Individual level intra-annual pattern of xylogenesis for red maple during 2017-2019. The points and lines correspond to the raw and fitted data using a generalized additive model, respectively. Individual trees are colour-coded (legend on the right). Zone widths for cell elongation (EZ), cell-wall thickening (WZ), and mature xylem cells (MZ) are displayed in the left, middle, and right panels, respectively.

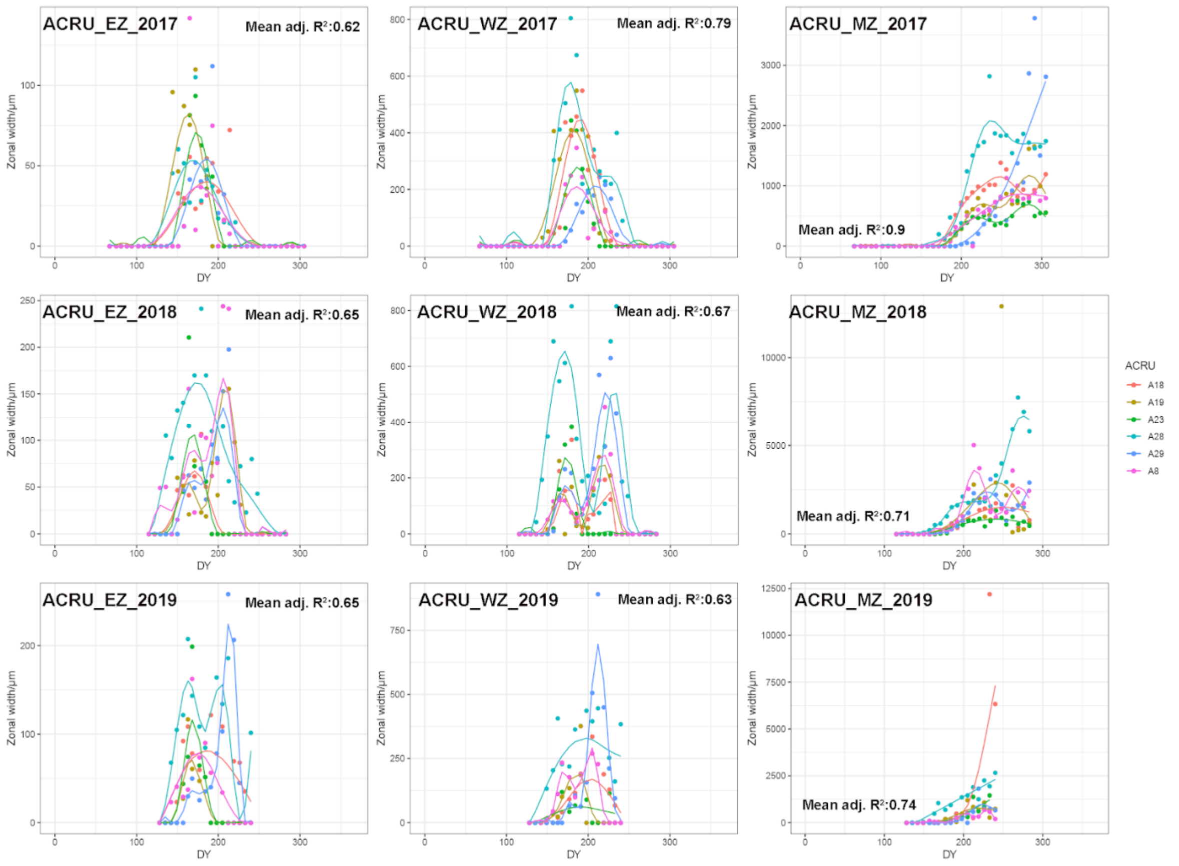

Fig. S4 Individual level intra-annual pattern of xylogenesis for white pine during 2017-2019. The points and lines correspond to the raw and fitted data using a generalized additive model, respectively. Individual trees are colour-coded (legend on the right). Zone widths for cell elongation (EZ), cell-wall thickening (WZ), and mature xylem cells (MZ) are displayed in the left, middle, and right panels, respectively.

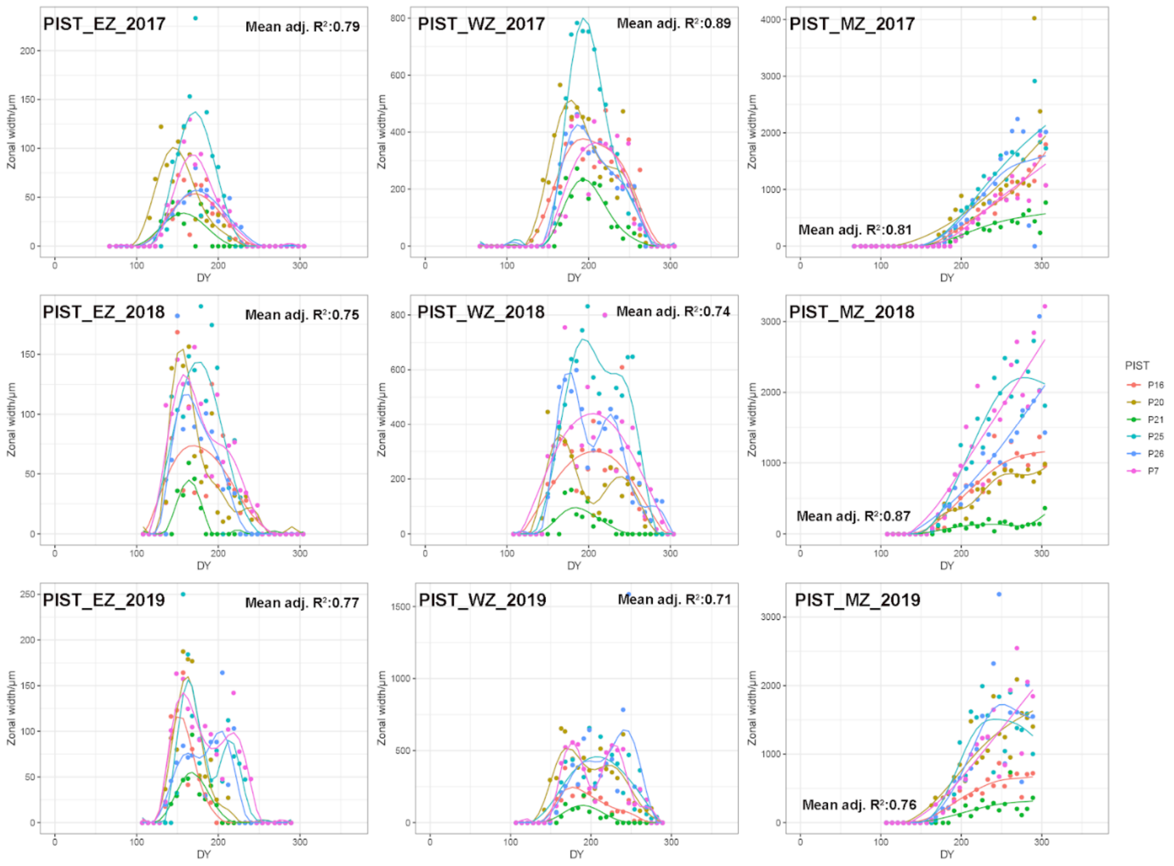

Fig. S5 Comparison of weekly precipitation of the study years against multiple year mean. DY: day of year. Prep_2017: weekly precipitation in 2017, Prep_MYM: weekly precipitation of multiple year mean.

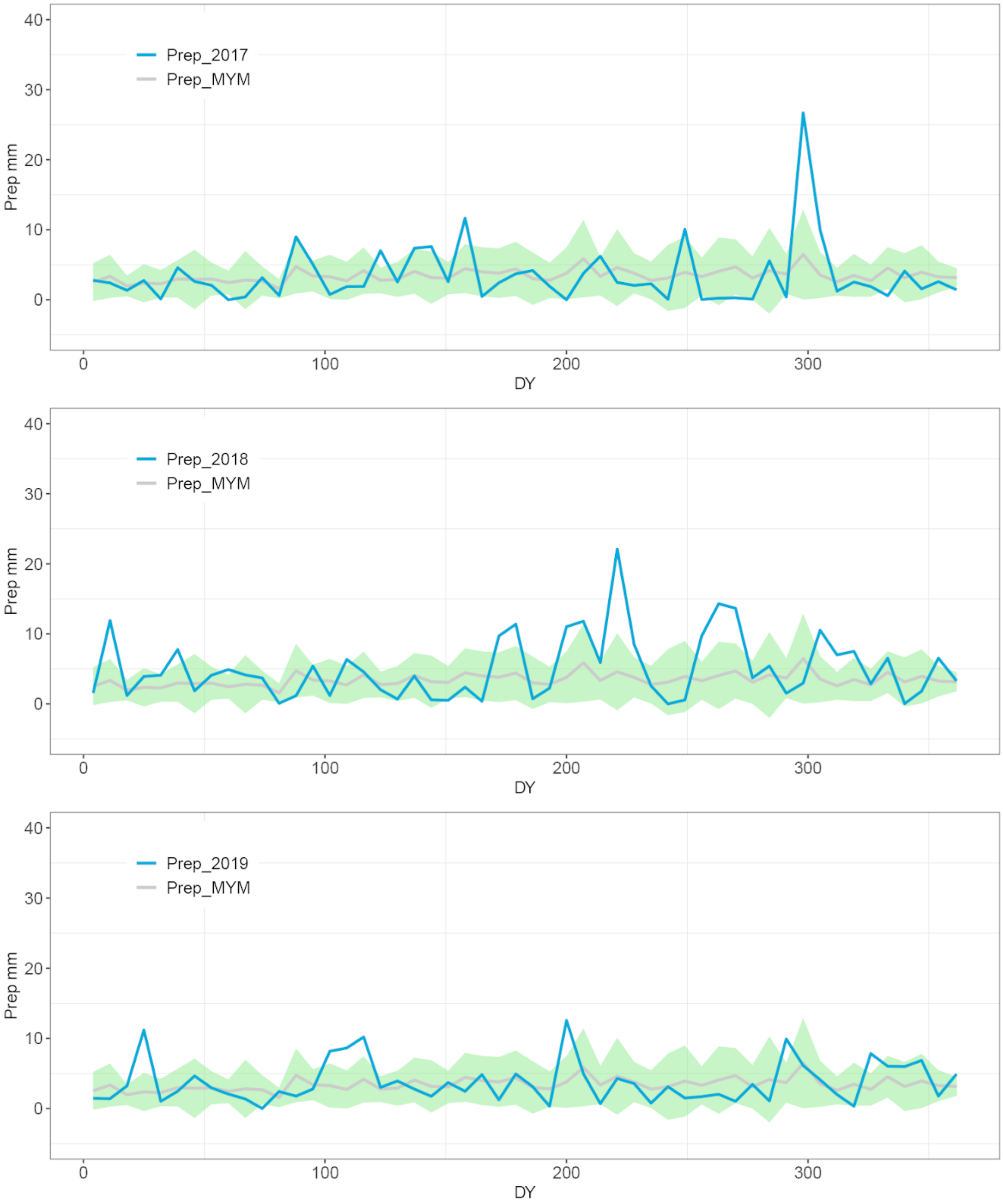

Fig. S6 Correlations between mean annual diameter at breast height (DBH) and mean annual ring width of individual trees for (a) red oak (QURU), (b) red maple (ACRU) and (c) white pine (PIST).

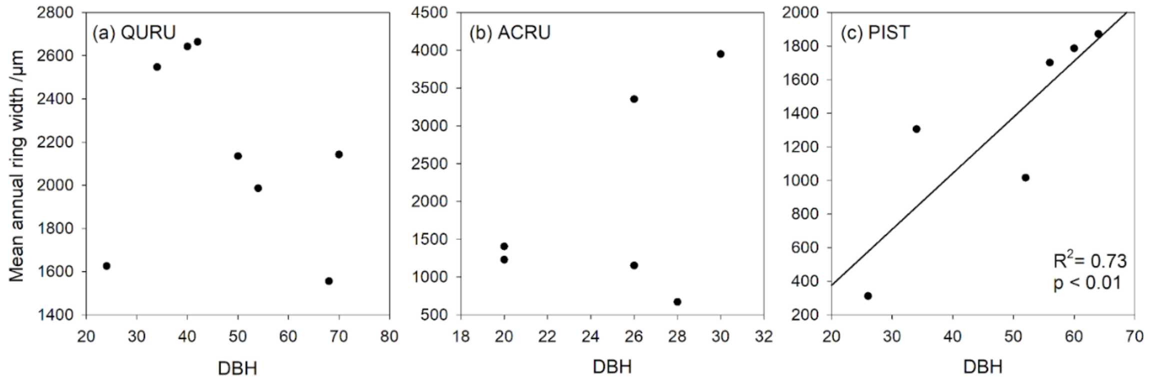

Fig. S7 Seasonal dynamics of stem NSC for (a) red oak (QURU), (b) red maple (ACRU) and (c) white pine (PIST) of individuals from 2017 to 2019.

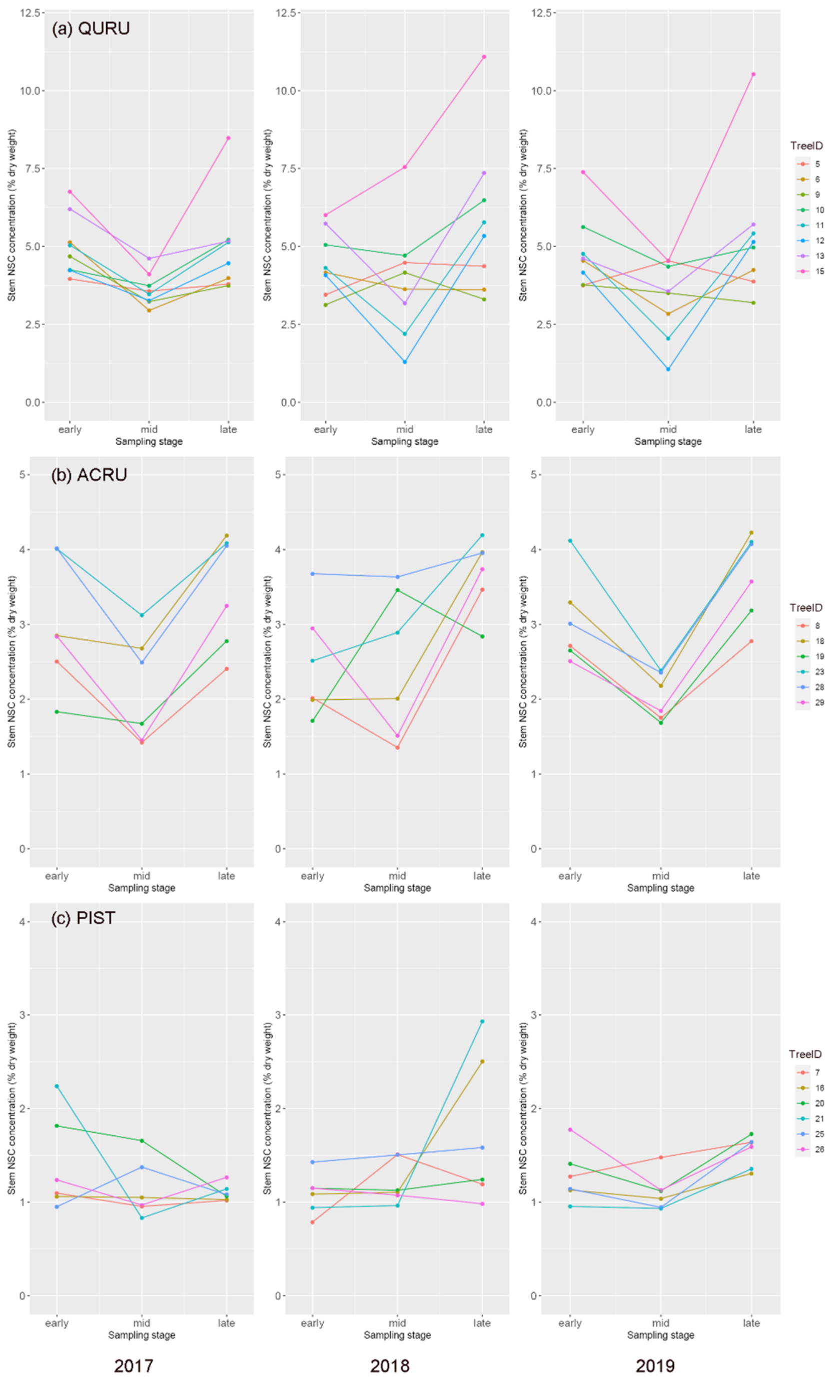

Fig. S8 Dates of enlargement cessation of individuals (cE_date) for (a) red oak (QURU), (b) red maple (ACRU) and (c) white pine (PIST).

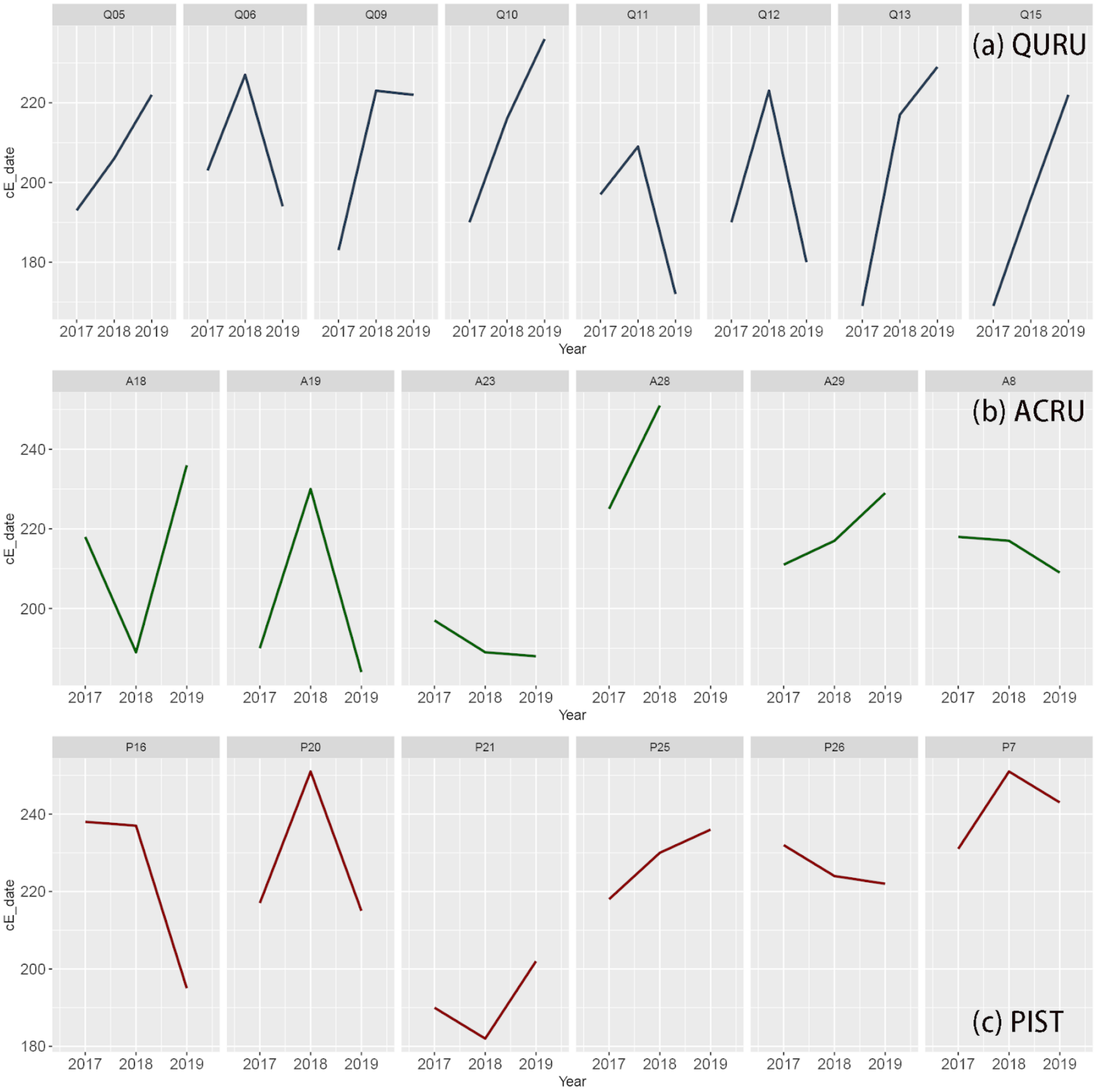

Fig. S9 The interannual patterns of mean ring width of red oak, maple and white pine from 2016 to 2019. QURU: red oak, ACRU: red maple, PIST: white pine; rw_p: mean ring width calculated from standardization data for the previous year, rw_c: mean ring width calculated from the xylogenesis observation of the current year. Approach to estimate rw_p and rw_c can be found in Notes S1.

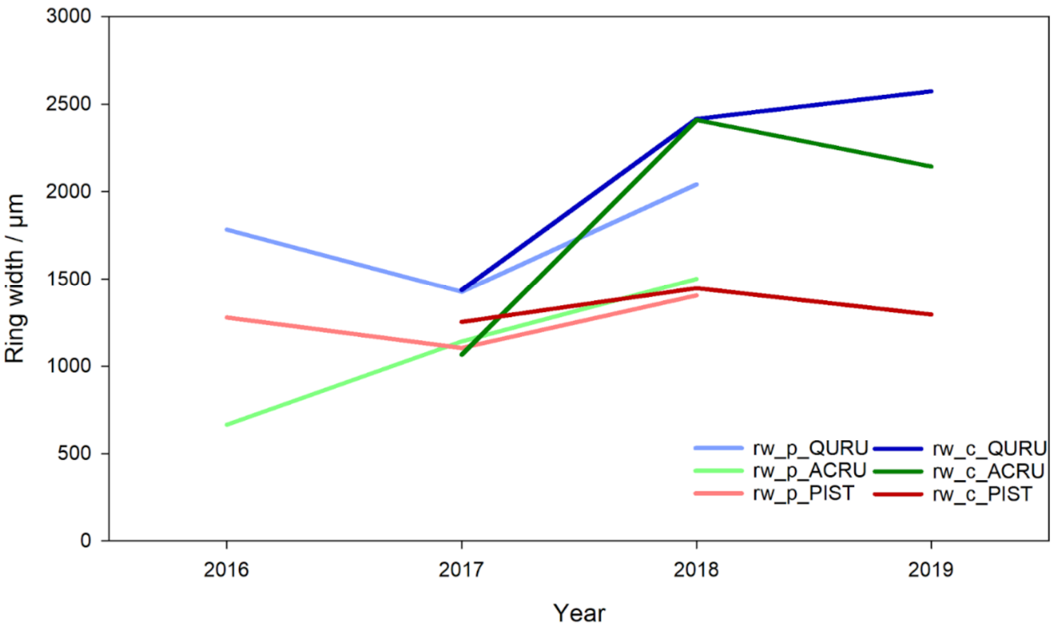

Table S1 Summary of important growth indexes for red oak (QURU), red maple (ACRU) and white pine (PIST). G_{max}: maximum width of enlarging xylem, µm, G_{mean}: mean width of enlarging xylem, µm, G_{len}: days of enlarging period, days, RW: ring width at the end of cell enlargement phase, µm.

Year	$G_{max}(\mu \pm \sigma)$	$G_{mean}(\mu \pm \sigma)$	$G_{len}(\mu \pm \sigma)$	RW$(\mu \pm \sigma)$
QURU				
2017	122.62±32.05	63.56±14.22	78.38±13.87	1437.08±327.06
2018	114.40±34.08	58.29±11.02	99.86±9.19	2537.66±446.75
2019	140.02±35.60	76.12±14.66	100.86±22.74	2645.00±976.4
ACRU				
2017	56.24±17.44	30.17±9.78	61.50±14.82	1066.00±482.82
2018	131.79±38.71	63.20±15.50	70.50±30.27	2409.27±1863.19
2019	114.05±64.01	54.07±17.60	59.80±25.70	2097.20±3151.72
PIST				
2017	79.29±38.13	39.63±18.69	96.33±19.19	1253.70±419.74
2018	110.83±42.94	54.49±19.28	92.83±30.71	1447.13±870.05
2019	121.42±40.02	59.00±19.37	83.33±18.34	1296.35±675.68

Table S2 Linear mixed models to evaluate the responses of the weekly variations of enlargement zone, against day length (DL), air temperature (Ta) and precipitation (Prep) for red oak (QURU) during 2017-2019. The corrected Akaike's Information Criterion increments (ΔAIC_c) for each model are shown with respect to that of the model with lowest score (the best fitted model). $\Delta AIC_c > 2$ are considered to be significant.

QURU		Variations of enlargement zone		
Models	Fixed effects	ΔAICc	Marginal R^2	Conditional R^2
M1	None	574.30	0.00	0.0051
M2	Year	533.00	0.059	0.064
M3	DL	258.40	0.36	0.37
M4	Ta	573.20	0.0043	0.0094
M5	Prep	572.20	0.0058	0.11
M6	DL * Year	219.70	0.40	0.40
M7	Ta * Year	534.90	0.062	0.071
M8	Prep * Year	532.00	0.066	0.070
M9	DL * Year + Ta * Year	74.80	0.51	0.52
M10	DL * Year + Prep * Year	214.80	0.40	0.41
M11	Prep * Year + Ta * Year	533.50	0.069	0.074
M12	DL * Year + Ta * Year + Prep * Year	64.00	0.52	0.53
M13	DL * Ta * Year	19.50	0.55	0.56
M14	Prep * Ta * Year	535.90	0.071	0.076
M15	DL * Prep * Year	208.10	0.41	0.42
M16	DL * Ta * Prep * Year	0.00	0.57	0.58

Table S3 Linear mixed models to evaluate the responses of the weekly variations of enlargement zone, against day length (DL), air temperature (Ta) and precipitation (Prep) for red maple (ACRU) during 2017-2019. The corrected Akaike's Information Criterion increments (ΔAIC_c) for each model are shown with respect to that of the model with lowest score (the best fitted model). $\Delta AIC_c > 2$ are considered to be significant.

ACRU		Variations of enlargement zone		
Models	Fixed effects	ΔAICc	Marginal R^2	Conditional R^2
M1	None	249.50	0.00	0.05
M2	Year	189.50	0.12	0.17
M3	DL	95.30	0.28	0.33
M4	Ta	173.20	0.15	0.20
M5	Prep	249.30	0.0047	0.05
M6	DL * Year	56.40	0.34	0.39
M7	Ta * Year * Species	126.40	0.24	0.29
M8	Prep * Year	186.70	0.13	0.18
M9	DL * Year + Ta * Year	26.60	0.38	0.44
M10	DL * Year + Prep * Year	38.40	0.37	0.42
M11	Prep * Year + Ta * Year	127.80	0.24	0.29
M12	DL * Year + Ta * Year + Prep * Year	15.30	0.40	0.46
M13	DL * Ta * Year	4.40	0.41	0.47
M14	Prep * Ta * Year	125.10	0.25	0.30
M15	DL * Prep * Year * Species	42.40	0.37	0.42
M16	DL * Ta * Prep * Year	0	0.44	0.49

Table S4 Linear mixed models to evaluate the responses of the weekly variations of enlargement zone, against day length (DL), air temperature (Ta) and precipitation (Prep) for white pine (PIST) during 2017-2019. The corrected Akaike's Information Criterion increments (ΔAIC_c) for each model are shown with respect to that of the model with lowest score (the best fitted model). $\Delta AIC_c > 2$ are considered to be significant.

PIST		Variations of enlargement zone		
Models	Fixed effects	ΔAICc	Marginal R^2	Conditional R^2
M1	None	419.40	0.00	0.065
M2	Year	406.80	0.025	0.090
M3	DL	76.00	0.44	0.51
M4	Ta	329.4	0.15	0.21
M5	Prep	420.10	0.0022	0.067
M6	DL * Year	58.80	0.46	0.53
M7	Ta * Year	312.60	0.18	0.24
M8	Prep * Year	408.20	0.029	0.095
M9	DL * Year + Ta * Year	62.20	0.46	0.53
M10	DL * Year + Prep * Year	55.70	0.47	0.53
M11	Prep * Year + Ta * Year	314.40	0.18	0.25
M12	DL * Year + Ta * Year + Prep * Year	59.40	0.47	0.53
M13	DL * Ta * Year	10.40	0.50	0.58
M14	Prep * Ta * Year	317.80	0.18	0.25
M15	DL * Prep * Year * Species	52.80	0.47	0.54
M16	DL * Ta * Prep * Year	0.00	0.53	0.60

Table S5 All linear mixed models tested to evaluate the effect of the maximum enlargement zone width (G_{max}) or mean enlargement zone width (G_{mean}), and the duration of enlargement phase (G_{len}) on the annual ring width for the three species (red oak, red maple and white pine) during 2017-2019. The corrected Akaike's Information Criterion increments (ΔAIC_c) for each model are shown with respect to that of the model with lowest score (the best fitted model). $\Delta AIC_c > 2$ are considered to be significant.

		Annual ring width		
Models	Fixed effects	ΔAICc	Marginal R^2	Conditional R^2
M1	None	31.72	0.00	0.097
M2	Year	28.97	0.072	0.24
M3	Species	28.95	0.077	0.09
M4	G_{max}	28.95	0.083	0.21
M5	G_{max} * Species	33.98	0.14	0.20
M6	G_{max} * Year	30.13	0.12	0.29
M7	G_{max} * Species * Year	33.34	0.30	0.46
M8	G_{mean}	21.27	0.20	0.31
M9	G_{mean} * Species	25.93	0.25	0.37
M10	G_{mean} * Year	23.54	0.22	0.33
M11	G_{mean} * Species * Year	32.25	0.31	0.45
M12	G_{len}	14.95	0.31	0.45
M13	G_{len} * Species	1.65	0.50	0.62
M14	G_{len} * Year	12.43	0.36	0.59
M15	G_{len} * Species * Year	0.00	0.60	0.69
M16	G_{max} * G_{len}	18.00	0.32	0.53
M17	G_{max} * G_{len} * Species	9.19	0.55	0.61
M18	G_{max} * G_{len} * Year	15.12	0.42	0.67
M19	G_{max} * G_{len} * Species * Year	8.45	0.70	0.76
M20	G_{mean} * G_{len}	14.43	0.36	0.54
M21	G_{mean} * G_{len} * Species	6.93	0.56	0.64
M22	G_{mean} * G_{len} * Year	18.09	0.40	0.62
M23	G_{mean} * G_{len} * Species * Year	12.85	0.68	0.72

Table S6 Linear mixed models to evaluate the effect of the maximum enlargement zone width (G_{max}), the mean enlargement zone width (G_{mean}) and the duration of enlargement phase (G_{len}) on the annual ring width for red oak (QURU) during 2017-2019. The factor of the individual tree was considered as the random effect. The corrected Akaike's Information Criterion increments (ΔAIC_c) for each model are shown with respect to that of the model with lowest score (the best fitted model). $\Delta AIC_c > 2$ are considered to be significant.

QURU		Annual ring width		
Models	Fixed effects	ΔAICc	Marginal R^2	Conditional R^2
M1	None	19.55	0.00	0.00
M2	Year	10.08	0.38	0.50
M3	G_{max}	21.10	0.019	0.019
M4	G_{max} * Year	13.29	0.38	0.47
M5	G_{len}	3.33	0.53	0.70
M6	G_{len} * Year	0.00	0.60	0.77
M7	G_{max} * G_{len}	2.00	0.60	0.69
M8	G_{max} * G_{len} * Year	0.70	0.68	0.75
M9	G_{mean}	18.28	0.13	0.14
M10	G_{mean} * Year	9.98	0.46	0.49
M11	G_{max} * G_{len} * Year	0.49	0.61	0.81

Table S7 Linear mixed models to evaluate the effect of the maximum enlargement zone width (G_{max}), the mean enlargement zone width (G_{mean}) and the duration of enlargement phase (G_{len}) on the annual ring width for red maple (ACRU) during 2017-2019. The factor of the individual tree was considered as the random effect. The corrected Akaike's Information Criterion increments (ΔAIC_c) for each model are shown with respect to that of the model with lowest score (the best fitted model). $\Delta AIC_c > 2$ are considered to be significant.

ACRU		Annual ring width		
Models	Fixed effects	ΔAICc	Marginal R^2	Conditional R^2
M1	None	8.58	0.00	0.00
M2	Year	9.67	0.054	0.15
M3	G_{max}	9.89	0.053	0.20
M4	G_{max} * Year	11.05	0.16	0.36
M5	G_{len}	0.00	0.45	0.58
M6	G_{len} * Year	0.16	0.50	0.61
M7	G_{max} * G_{len}	3.81	0.42	0.51
M8	G_{max} * G_{len} * Year	3.86	0.53	0.59
M9	G_{mean}	7.47	0.17	0.39
M10	G_{mean} * Year	11.37	0.15	0.34
M11	G_{mean} * G_{len} * Year	5.50	0.49	0.54

Table S8 Linear mixed models to evaluate the effect of the maximum enlargement zone width (G_{max}), the mean enlargement zone width (G_{mean}) and the duration of enlargement phase (G_{len}) on the annual ring width for white pine (PIST) during 2017-2019. The factor of the individual tree was considered as the random effect. The corrected Akaike's Information Criterion increments (ΔAIC_c) for each model are shown with respect to that of the model with lowest score (the best fitted model). $\Delta AIC_c > 2$ are considered to be significant. It should be noted that there is a significant effect from the individual tree, i.e., the random effect, so we further built the models using the tree factor as the fixed effect and tested its contribution. The result was showed in Table S9.

PIST		Annual ring width		
Models	Fixed effects	ΔAICc	Marginal R^2	Conditional R^2
M1	None	5.33	0.00	0.68
M2	Year	7.29	0.00	0.66
M3	G_{max}	5.09	0.14	0.55
M4	G_{max} * Year	7.35	0.31	0.52
M5	G_{len}	5.60	0.08	0.63
M6	G_{len} * Year	5.75	0.22	0.61
M7	G_{max} * G_{len}	3.19	0.39	0.64
M8	G_{max} * G_{len} * Year	9.06	0.41	0.58
M9	G_{mean}	0.37	0.49	0.53
M10	G_{mean} * Year	0.00	0.59	0.59
M11	G_{mean} * G_{len} * Year	5.43	0.55	0.57

Table S9 Linear mixed models to evaluate the effect of the mean enlargement zone width (G_{mean}) and the individual tree (tree) on the annual ring width for white pine (PIST) during 2017-2019. The factor of year was considered as the random effect. The corrected Akaike's Information Criterion increments (ΔAIC_c) for each model are shown with respect to that of the model with lowest score (the best fitted model). $\Delta AIC_c > 2$ are considered to be significant.

PIST		Annual ring width		
Models	Fixed effects	ΔAICc	Marginal R^2	Conditional R^2
M1	None	15.18	0.00	0.00
M2	Tree	0.00	0.68	0.68
M3	G_{mean}	3.19	0.55	0.60
M4	G_{mean} * Tree	2.94	0.67	0.67

Notes S1 Quantification of annual ring width based on the standardization data

Since ring widths from the previous year were measured to standardize the data at the current year for each image, it is possible to calculate the ring width of 2016-2018 based on that. We then averaged the ring width of each image for each year from the three species. The mean ring width from the current year was quantified by summarizing the observations of xylogenesis from the images after the cessation of enlargement phase for each individual tree, as described in Section 2.2.2. In sum, we got the ring width observations from the standardization data of the previous year for the period of 2016 - 2018 (rw_p) and the xylogenesis data of the current year for the period of 2017 - 2019 (rw_c).
